# Editorial: PARP inhibitors resistance: new insights in ovarian cancer therapy

**DOI:** 10.3389/fonc.2025.1680969

**Published:** 2025-09-02

**Authors:** Oraianthi Fiste, Michalis Liontos, Sana Intidhar Labidi-Galy

**Affiliations:** ^1^ Oncology Unit, Third Department of Internal Medicine and Laboratory, National and Kapodistrian University of Athens, Sotiria General Hospital, Athens, Greece; ^2^ Department of Clinical Therapeutics, National and Kapodistrian University of Athens, Alexandra Hospital, Athens, Greece; ^3^ Department of Oncology, Hôpitaux Universitaires de Genève, Geneva, Switzerland; ^4^ Department of Medicine, Division of Oncology, Faculty of Medicine, University of Geneva, Swiss Cancer Center Leman, Geneva, Switzerland

**Keywords:** ovarian cancer, PARP inhibitors, resistance mechanisms, biomarkers, homologous recombination deficiency (HRD), BRCA 1/2 mutations

Ovarian cancer remains the leading cause of gynecological cancer-related mortality worldwide, largely due to the absence of a screening program and its subsequent late diagnosis. The vast majority of patients are indeed diagnosed with advanced stage disease, for which (neo)-adjuvant platinum-based chemotherapy and optimal debulking surgery represent the standard of care.

In recent years, the therapeutic landscape of ovarian cancer has nevertheless undergone a dramatic shift with the implementation of targeted therapies, including the antiangiogenic monoclonal antibody bevacizumab and the orally administered poly (ADP-ribose) polymerase inhibitors (PARPi). By harnessing the principle of synthetic lethality in tumors characterized by homologous recombination deficiency (HRD), especially those harboring BRCA1/2 genomic alterations, PARPi have provided meaningful clinical benefits. Of note, 25% of epithelial ovarian cancer cases are attributed to BRCA1/2 mutations, whereas almost 50% exhibit HRD.

Yet, resistance emergence is rather inevitable leading to incurable disease recurrence and unfavorable prognosis. In light of this therapeutic challenge the Research Topic *“PARP inhibitors resistance: new insights in ovarian cancer therapy*” of Frontiers in Oncology comprises four insightful original articles that not only enhance our understanding of PARPi mechanism of action but also elucidate the complexity of the underlying resistance mechanisms, as well as explore emerging translational strategies in order to overcome these hurdles and improve patients’ outcomes.

In their comprehensive review, Kulkarni et al. discusses the role of PARPi in the treatment of high-grade serous ovarian cancer, analyzing the efficacy results of pivotal clinical trials of PARPi including but not limited to olaparib, rucaparib, and niraparib, and commenting on their safety profile. The authors later on provide a detailed description of primary and acquired resistance mechanisms, the present therapeutic algorithm for patients with progression on PARPi based on platinum-free interval, as well as the urgent need for potential predictive biomarkers of PARPi sensitivity and resistance. Last but not least, current and investigational approaches, like immunotherapy, antibody drug conjugates, and additional synthetic lethal partners are being thoroughly addressed.

Complementing the previous review, Collet et al. reaffirms the foundational role of BRCA1/2 alterations in driving sensitivity to PARPi but also highlights the importance of reversion mutations of these genes in PARPi resistance. The authors deepen the narrative by addressing the promising role of liquid biopsies, particularly circulating tumor DNA, as a “real-time” surrogate biomarker of treatment response. Equally significant, in conclusion they explore preclinical and clinical data of emerging therapeutic strategies in order to delay if not overcome PARPi resistance.

The original study of Kim et al. focused on the immunohistochemical expression of RAD51 as a functional and cost-effective biomarker of homologous recombination (HR) restoration for PARPi resistance. Despite its retrospective nature and the limited sample size, the present study is of great importance as it underscores the comparative advantage of RAD51 assessment in reflecting the dynamic HRD status during PARPi treatment, compared to the commercially available methodologies, like loss of heterozygosity (LOH), telomeric allelic imbalance (TAI), and/or large-scale state transitions (LST).

Finally, the experimental study of Berckmans et al. utilized ovarian cancer cell cultures (*in vitro*) and mice xenograft models (*in vivo*) to evaluate the potential of the tumor treating fields (TTFields) in inducing an HRD-like phenotype when combined with carboplatin or PARPi. Regarding their mechanism of action, TTFields use low-intensity, intermediate-frequency alternating electric fields in order to disrupt cancer cell division by interfering with mitosis. The investigators concluded that this novel, localized, non-invasive anticancer treatment could sensitize resistant cancer cells to overcome platinum- and/or PARPi resistance, thus merits validation through larger-scale prospective studies.

Taken together, the aforementioned contributions provide a cohesive and future-oriented perspective on the biology and clinical translation of PARPi resistance. It has been our privilege to curate this Research Topic, which we sincerely hope will serve not only as a valuable scientific resource but also as a catalyst for future research, ultimately advancing the pursuit of personalized thus optimal treatment strategies for ovarian cancer patients.

